# Socioeconomic outcomes in very preterm/very low birth weight adults: individual participant data meta-analysis

**DOI:** 10.1038/s41390-025-04082-1

**Published:** 2025-05-03

**Authors:** Yanlin Zhou, Marina Mendonça, Nicole Tsalacopoulos, Peter Bartmann, Brian A. Darlow, Sarah L. Harris, John Horwood, Lianne J. Woodward, Peter J. Anderson, Lex W. Doyle, Jeanie L. Y. Cheong, Eero Kajantie, Marjaana Tikanmäki, Samantha Johnson, Neil Marlow, Chiara Nosarti, Marit S. Indredavik, Kari Anne I. Evensen, Katri Räikkönen, Kati Heinonen, Sylvia van der Pal, Dieter Wolke

**Affiliations:** 1 Department of Psychology, University of Warwick, Coventry, United Kingdom; 2 School of Psychology and Vision Sciences, University of Leicester, Leicester, United Kingdom; 3 Department of Population Health Sciences, University of Leicester, Leicester, United Kingdom; 4 School of Psychology Sciences, Monash University, Melbourne, VIC Australia; 5 Department of Neonatology and Paediatric Intensive Care, University Hospital Bonn, Children’s Hospital, Bonn, Germany; 6 Department of Paediatrics, University of Otago Christchurch, Christchurch, New Zealand; 7 Department of Psychological Medicine, University of Otago Christchurch, Christchurch, New Zealand; 8 Canterbury Child Development Research Group, Faculty of Health, University of Canterbury, Christchurch, New Zealand; 9 Clinical Sciences, Murdoch Children’s Research Institute, Melbourne, VIC Australia; 10 Department of Obstetrics, Gynaecology and Newborn Health, The University of Melbourne, Melbourne, VIC Australia; 11 Newborn Research Centre, The Royal Women’s Hospital, Melbourne, VIC Australia; 12 Department of Paediatrics, The University of Melbourne, Melbourne, VIC Australia; 13 Clinical Medicine Research Unit, MRC Oulu, Oulu University Hospital and University of Oulu, Oulu, Finland; 14 Population Health Unit, Finnish Institute for Health and Welfare, Helsinki, Finland; 15 Children’s Hospital, Helsinki University Hospital, University of Helsinki, Helsinki, Finland; 16 Department of Clinical and Molecular Medicine, Norwegian University of Science and Technology, Trondheim, Norway; 17 Elizabeth Garrett Anderson Institute for Women’s Health, University College London, London, United Kingdom; 18 Department of Child and Adolescent Psychiatry, Institute of Psychiatry, Psychology and Neuroscience, King’s College London, London, United Kingdom; 19 Centre for the Developing Brain, School of Biomedical Engineering & Imaging Sciences, King’s College London, London, United Kingdom; 20 Children’s Clinic, St. Olavs Hospital, Trondheim University Hospital, Trondheim, Norway; 21 Department of Rehabilitation Science and Health Technology, Oslo Metropolitan University, Oslo, Norway; 22 Department of Psychology and Logopedics, University of Helsinki, Helsinki, Finland; 23 Department of Obstetrics and Gynecology, Helsinki University Hospital and University of Helsinki, Helsinki, Finland; 24 Welfare Sciences/Psychology, Tampere University, Tampere, Finland; 25 Department of Child Health, Netherlands Organization for Applied Scientific Research TNO, Leiden, the Netherlands; 26 Division of Health Sciences, Warwick Medical School, University of Warwick, Coventry, United Kingdom

## Abstract

**Background:**

Very preterm (VPT; <32 weeks) or very low birth weight (VLBW; <1500 g) birth is associated with socioeconomic disadvantages in adulthood; however, the predictors of these outcomes remain underexplored. This study examined socioeconomic disparities and identified neonatal and sociodemographic risk factors among VPT/VLBW individuals.

**Methods:**

A one-stage individual participant data meta-analysis was conducted using 11 birth cohorts from eight countries, comprising 1695 VPT/VLBW and 1620 term-born adults aged 18–30 years.

**Results:**

VPT/VLBW adults had lower odds of higher educational attainment (0.40[0.26–0.59]), remaining in education (0.63[0.47–0.84]) or paid work (0.76[0.59–0.97]), and higher odds of receiving social benefits (3.93[2.63–5.68]) than term-borns. Disparities in education and social benefits persisted after adjusting for age, sex, and maternal education, even among those without neurosensory impairments (NSI). Among VPT/VLBW adults, NSI significantly impacted all socioeconomic outcomes, increasing the odds of receiving social benefits 6.7-fold. Additional risk factors included medical complications, lower gestational age and birth weight, lower maternal education, younger maternal age, and non-white ethnicity.

**Conclusions:**

NSI is the strongest risk factor for adulthood socioeconomic challenges in the VPT/VLBW population. Mitigating these disparities may require improved neonatal care to reduce NSI prevalence and targeted social and educational support for VPT/VLBW individuals.

**Impact:**

- Very preterm or very low birth weight (VPT/VLBW) birth is associated with socioeconomic disadvantages in adulthood, including lower educational attainment, lower employment rates, and a higher need for social benefits compared with individuals born at term.
- Neurosensory impairments are strongly associated with adverse socioeconomic outcomes among VPT/VLBW adults, while lower gestational age, lower birth weight, and sociodemographic disadvantages serve as additional risk factors.
- Early interventions in the NICU that reduce medical complications, along with enhanced educational support throughout childhood, may help mitigate long-term socioeconomic disparities for individuals born VPT/VLBW.

## Introduction

Approximately 1 to 2 per hundred infants are born very preterm (VPT; <32 weeks’ gestation) or with very low birth weight (VLBW; <1500 g).^1,2^ Advances in obstetric and neonatal intensive care since the 1960s have markedly increased the survival rates of VPT/VLBW infants,^3,4^ resulting in a growing number of survivors reaching adulthood than ever before. Individuals born VPT/VLBW are at increased risk of physical health, cognitive, mental health, schooling, and social development difficulties.^2,5–9^ These difficulties often extend into adulthood affecting their socioeconomic outcomes, including educational attainment, workforce participation, receipt of social benefits, and independent living.^2,10^ Yet, while previous individual studies^11–13^ and two meta-analyses^14,15^ have reported socioeconomic disparities between VPT/VLBW survivors and term-born adults, the early factors that may contribute to their socioeconomic outcomes have rarely been studied. Understanding these long-term challenges is crucial, as socioeconomic outcomes are not only important determinants of an individual’s health, longevity, and quality of life, but also key markers of societal integration, contributions, and overall wellbeing.^16,17^

### Inconsistent group differences in individual studies

Differences in educational attainment, workforce participation, and receipt of social benefits between VPT/VLBW and term-born or normal-birth-weight peers have been widely examined. Scandinavian registry-based studies have consistently shown that VPT/VLBW individuals are less likely to achieve higher educational qualifications or participate in the workforce, and are more likely to earn lower incomes and receive social benefits.^11,12,18–21^ Similarly, two British cohorts reported that VPT/VLBW had lower educational attainment in young adulthood and lower wealth levels (i.e., family income, social class, housing tenure, employment status, and income) by age 42.^22^ However, findings from the Ontario Child Health Study suggest a more complex picture. Among 166 extremely low birth weight (ELBW; <1000 g) survivors, educational levels young adulthood (22–25 years) and early thirties (29–36 years) was comparable to that of their term-born peers.^23,24^ But these ELBW individuals were less likely to be employed, work full-time, or earn higher incomes, and more likely to receive social benefits in their thirties.^24^

Studies on independent living, usually defined as residing away from or having ever left the parental home, have also shown mixed findings. In a Canadian cohort, ELBW adults showed no significant differences from normal-birth-weight peers in independent living in their early twenties^23^ or thirties.^24^ Similarly, a Danish study found no significant differences between VPT and term-born individuals at ages 27–29, with over 90% having left their parental home.^20^ In contrast, a Swedish national cohort study found that VPT adults (23–29 years) were more likely to live with their parents than same-age, same-sex term-born peers.^12^ The Helsinki Study reported that VLBW adults lagged behind term-born peers in leaving their parental home during their late teens and early twenties.^25^ A Northern Finland birth cohort also showed fewer preterm-born young adults had ever lived independently, though this difference was not significant after adjusting for age at assessment.^26^

VPT/VLBW infants are at a significantly higher risk of neurosensory impairments (NSI), including cerebral palsy and severe hearing, vision, or cognitive impairments, which are also strongly associated with socioeconomic outcomes in adulthood.^27,28^ The inclusion or exclusion of individuals with NSI may contribute to variations in study findings. For example, some research has shown that differences in employment and social benefits between ELBW and term-born peers became non-significant after excluding individuals with NSI.^23,24^ This suggests that unfavourable socioeconomic outcomes in the VPT/VLBW population may partly reflect the long-term impact of NSI.^12,18,29^ However, even among individuals without NSI, studies have reported persistent socioeconomic disparities. For example, VLBW adults without NSI show lower educational attainment and programme enrolment compared to normal-birth-weight controls,^29^ while VPT adults show lower educational attainment, reduced income, and higher reliance on social benefits.^18^ These findings, however, are based on individual studies with small sample sizes. Larger studies and meta-analyses are needed to evaluate the extent to which specific factors such as NSI, age, and sex, may partly explain socioeconomic disparities between VPT/VLBW and term-born adults.

### Insights and limitations of previous meta-analyses

The individual studies mentioned above are often not directly comparable due to differences in study characteristics (e.g., country, design, sample size, follow-up attrition), sample characteristics (e.g., degree of prematurity, age at assessment, inclusion of participants with NSI), socioeconomic outcome indicators (e.g., highest educational level, university completion, years of education), and analytical methods (e.g., Chi-square test, logistic regression). Two meta-analyses have provided broader insights into the socioeconomic challenges of adults born preterm, categorised by degrees of birth weight and gestational age. Lambiris et al. analysed aggregate data from 15 studies and found that each standard deviation increase in birth weight (approximately 500 g) corresponded to a 2.8% increase in annual earnings.^15^ Bilgin et al.’s meta-analysis of 23 registry and cohort studies examined four socioeconomic indicators, showing that preterm birth or low birth weight was negatively associated with educational attainment and employment status and positively associated with receiving social benefits.^14^ A dose-response relationship was observed, with greater disadvantages for those born VPT than moderate-to-late preterm (32–36 gestational weeks). However, results on independent living were inconclusive, possibly due to variability in assessments and cross-country differences in social support systems.^14^ While these meta-analyses highlight association between VPT/VLBW birth and adulthood socioeconomic outcomes, their conclusions may be limited by publication bias and high heterogeneity among studies.^14,15^

Another key limitation of previous aggregate meta-analysis^2,30^ is their inability to capture how neonatal and psychosocial factors associate with adulthood socioeconomic outcomes. Among VPT/VLBW adults, previous meta-analyses suggest that lower gestational age and birth weight (both as continuous measures) are associated with lower adulthood IQ,^5^ while lower gestational age is also related to fewer romantic and sexual experiences in early adulthood.^31^ Moreover, two of the most common serious medical complications of preterm birth—intraventricular haemorrhage (IVH) and bronchopulmonary dysplasia (BPD)—are associated with increased mortality, respiratory morbidity, neurodevelopmental challenges, and lower IQ scores in adulthood.^5,32,33^ Among sociodemographic factors, higher parental education, older age, and male sex have been associated with higher adulthood socioeconomic outcomes.^12,18,34^ While these neonatal and sociodemographic factors have been examined in individual studies, they have not been systematically assessed in meta-analyses of adulthood socioeconomic outcomes among the VPT/VLBW population. This gap is largely due the lack of access to individual-level data in aggregate meta-analyses. An individual participant data (IPD) meta-analysis can overcome this limitation, allowing to investigate how neonatal, health, and sociodemographic factors are associated long-term socioeconomic outcomes in the VPT/VLBW population.

### The current study

In this study, we harmonised data from two international consortia of VPT/VLBW birth cohorts with term-born peers as controls and applied IPD meta-analysis to address two key objectives. First, we assessed differences in adult socioeconomic outcomes—educational attainment, workforce participation, social benefits, and independent living—between VPT/VLBW and term-born adults. These indicators were selected based on the recommendations of Common Core Assessments of the adults born preterm^35^ and a previous aggregate meta-analysis,^14^ covering key domains of the transition to adulthood, including education, financial stability (economically active, employment, social benefits), and functional independence (independent living). Second, we examined how neonatal and health factors (e.g., gestational age, birth weight, multiple birth, medical complications, NSI) and sociodemographic factors (age, sex, maternal education, maternal age at birth, ethnicity) are associated with socioeconomic outcomes within the VPT/VLBW population.

## Methods

### Protocol and registration

This study employs an IPD meta-analysis approach, which involves obtaining, harmonising, and analysing raw individual participant-level data. Recognised as the gold standard in systematic review methods,^36^ IPD meta-analysis improves data integration across studies, increases sample sizes, and enhances statistical power. It also enables adjustment for confounding factors when comparing VPT/VLBW and term-born adults and facilitates the examination of predictors of socioeconomic outcomes within the VPT/VLBW population.

This study was registered on PROSPERO (CRD42023432759) and followed the Preferred Reporting Items for Systematic Reviews and Meta-analyses (PRISMA) reporting guideline for IPD meta-analysis.^37^

### Study selection and inclusion criteria

The eligible study cohorts were identified from the Research on European Children and Adults Born Preterm (RECAP-preterm^38^) and the Adults Born Preterm International Collaboration (APIC) consortia.^35^ Inclusion criteria were: (1) longitudinal cohort studies involving VPT/VLBW ( < 32 weeks’ gestation and/or <1500 g birth weight); (2) a comparison control group of term-born ( ≥ 37 weeks) and/or normal birth weight ( > 2499 grams) individuals; and (3) assessment of at least one adulthood socioeconomic outcome ( ≥ 18 years). The Project on Preterm and Small-for-Gestational-Age Infants (POPS) cohort from the Netherlands included only VPT/VLBW participants but had a relatively large sample size.^39^ Therefore, in the primary analysis, it was included only in the assessment of predictors of socioeconomic outcomes among VPT/VLBW adults; however, sensitivity analyses with POPS are available in Table S3.

**Fig. 1:**
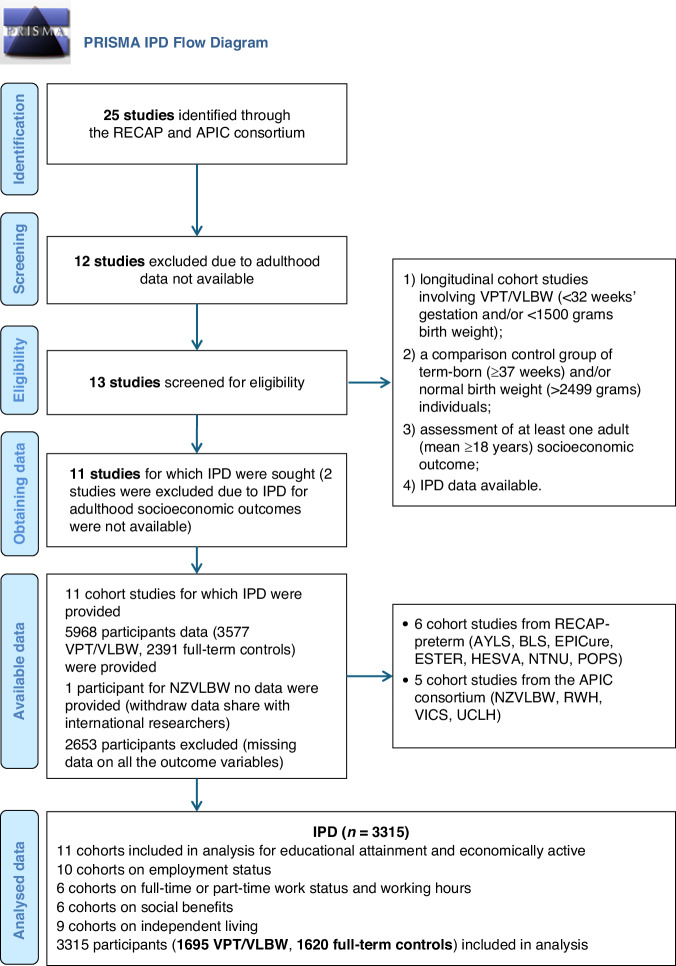
Flow diagram of studies included in the individual participant data (IPD) meta-analyses. Note. RECAP Research of European Children and Adults Born Preterm, APIC Adult Born Preterm International Collaboration, AYLS Arvo Ylppö Longitudinal Study, BLS Bavarian Longitudinal Study, ESTER The Preterm Birth and Early Life Programming of Adult Health and Disease Study, EPICure EPICure study, HESVA Helsinki Study of Very Low Birth Weight Adults, NTNU Norwegian University of Science and Technology Low Birth Weight in a Lifetime Perspective study, NZVLBW New Zealand Very Low Birth Weight Study, POPS Project on Preterm and Small for Gestational Age Infants, RWH Royal Women’s Hospital Study, UCLH University College London Hospital Cohort Study, VICS Victorian Infant Collaborative Study.

A total of 11 eligible cohorts (see Fig. 1) were identified—6 from RECAP-preterm and 5 from the APIC consortium—spanning eight countries: Finland, Norway, Ireland, the UK, the Netherlands, Germany, Australia, and New Zealand. All studies received country-specific ethical approvals and adhered to the Declaration of Helsinki. All participants provided written informed consent, except for one individual in the New Zealand cohort who withdrew permission to share data internationally; their data was excluded from this IPD meta-analysis.

### Risk of bias assessment

The risk of bias in the included cohorts was assessed using the Newcastle-Ottawa Quality Assessment Form for Cohort Studies.^40^ This tool evaluates study quality across three domains: selection of exposed and non-exposed groups (four items, scored 0–4), comparability of groups (one item, scored 0–2), and outcome assessment (three items, scored 0–3). Study quality was independently assessed by two reviewers, with any discrepancies resolved through discussion or consultation with a third reviewer. The detailed risk of bias assessment for each cohort, including individual criteria and assigned scores, is presented in Supplementary Table S1. Each cohort can achieve a maximum score of 9, categorised as “poor quality” (0–3 points), “fair quality” (4–6 points), and “good quality” (7–9 points) accordingly.^41^

### Data extraction and harmonisation

Data extracted from the selected 11 studies included: cohort information (country, starting year, sample size for current analysis, attrition rates), demographics (age at assessment, sex at birth), neonatal and health factors (birth weight, gestational age, head circumference, medical complications, NSI), maternal sociodemographic factors (maternal education, maternal age at birth, and ethnicity), and socioeconomic outcome (educational attainment, workforce participation, receipt of social benefits, and independent living). Raw data from all cohorts were harmonised, and study investigators were contacted to clarify unclear coding or address incomplete information during harmonisation. The harmonisation variables are available on the RECAP-Preterm website.

#### Socioeconomic outcomes

Educational attainment (i.e., the highest level of education completed by participants) was harmonised according to the International Standard Classification of Education (ISCED) 2011-codes^42^ into three categories: Low (ISCED 0–2, equivalent to secondary education or less), Middle (ISCED 3–5, covering upper secondary to short post-secondary education), and High (ISCED 6–8, referring to university education or equivalent).

Workforce participation was evaluated using four variables: (a) economically active (i.e., whether the participant was part of the workforce at the assessment time, with ‘0’ indicating economically inactive, in education/training, unemployed/no occupation, and ‘1’ indicating economically active, i.e., currently in paid work); (b) employment status, with three categories (‘0’ unemployed/unpaid work/social security, ‘1’ in full-time education/training, ‘2’ in paid work); (c) for those in paid work, work status was classified as either ‘0’ for part-time (<35 h per week) or ‘1’ for full-time (≥35 h per week) employment; and (d) working hours, assessed by the total number of weekly working hours.

Receipt of social benefits was harmonised based on whether participants or their household received any form of government social welfare subsidy for people in need (0 = no, 1 = yes). This included benefits such as disability allowance or financial assistance for inability to work, while other benefits such as child allowance was not included.

Independent living was defined as whether the participant resided independently from parents or protected accommodation (0 = not independent, 1 = independent living).

#### Neonatal, health, and sociodemographic factors

To assess within-group variability and potential dose-response relationships with outcomes in the VPT/VLBW population, gestational age was recorded as completed weeks and birth weight (in grams) was standardised to *z*-scores using the Fenton growth chart, adjusted for gestational age and sex.^43^ Head circumference at birth (in centimetres), and multiple birth status (0 = singleton, 1 = multiple) were also included. The presence of IVH (grades 1–4) and BPD—defined as requiring supplemental oxygen for ≥28 days after birth or at 36 weeks’ postmenstrual age^44^—were harmonised as binary variables within the VPT/VLBW group. NSI was defined as one or more of the following: visual impairment (blindness or low vision), uncorrected hearing loss, non-ambulatory cerebral palsy (wheelchair-bound or bedridden), and intellectual disability (IQ < 70). NSI presence was then summarised into a binary variable (0 = without NSI, 1 = with any NSI). Participants with missing data for NSI were assumed to be without impairment, given that the missing data for NSI (*n* = 118, 3.6% of the total sample) included 100 cases from the term-born control group, where NSI prevalence is low. To assess the impact of this assumption, we conducted a sensitivity analysis comparing results under three approaches, assuming participants with missing values did not have NSI, using raw data, and applying multiple imputation for missing data. As shown in Table S4, the results remained consistent across methods.

Maternal education was harmonized into low, middle, and high levels based on the ISCED criteria,^42^ using the highest maternal educational level recorded at birth, or if unavailable, from follow-up data during the participant’s childhood. Maternal age at birth was included as a continuous variable. Ethnicity was categorized into white or non-white groups based on maternal or child ethnicity data.

### Statistical analysis

IPD meta-analysis can be performed using either a two-stage or a one-stage approach.^45^ In the two-stage approach, individual studies are analysed separately and the aggregate results are then combined using a standard meta-analysis model. The one-stage approach integrates and analyses the IPD simultaneously using a generalised linear mixed model that accounts for participants clustering within studies and between-study heterogeneity. The one-stage approach is recommended when most included studies have small sample sizes, as it provides more exact likelihood estimation.^46^

All analyses were conducted in R (version 3.6.1) using mixed models to account for study-level variability. First, to examine the association between VPT/VLBW status and outcomes, both random-intercept models (accounting for study-level baseline differences) and random-slope models (allowing group effects to vary across studies) were tested. The heterogeneity index (τ²) were reported, with values closer to zero indicating less variabilities across studies.^45^ Model selection was based on the Likelihood Ratio Test (LRT), where a significant *p* value indicated a better fit for the random slope model. Outcome-specific models included ordinal regression for educational attainment (*clmm2* of the *ordinal* package), multinomial logistic regression for employment status (*mclogit* package), linear regression for working hours (*lmer* in the *lme4* package), and logistic regression for binary outcomes such as social benefits and independent living (*glmer* in the *lme4* package). Analyses included unadjusted effects of VPT/VLBW, adjusted for age at assessment, sex, and maternal education, as well as sensitivity analyses excluding participants with NSI. Results were reported as odds ratios (ORs) with 95% confidence intervals (CIs) for categorical outcomes and unstandardized estimates (B) with 95% CIs for continuous outcomes.

Second, to examine how neonatal, health, and sociodemographic factors associate with socioeconomic outcomes within the VPT/VLBW population, univariable and multivariable regression models were conducted. Predictors were modelled as fixed effects with random intercepts. Missing data were handled using multiple imputation by chained equations (*mice* package) in the multivariable analyses. For easier interpretation and comparison of effect sizes across factors, ORs and B values were converted to Cohen’s *d* (0.2 = small, 0.5 = medium, 0.8 = large).^47^

## Results

### Study and participant characteristics

Table 1 summarises the characteristics of each cohort, including country, birth year, age at assessment, sample size, sex distribution, maternal education levels, and available socioeconomic indicators. The pooled data comprised 1695 VPT/VLBW and 1620 term-born control participants born between 1977 and 1995, with a mean age ranging from 18 to 30 years. All cohorts provided data on educational attainment and economically active status, 10 cohorts on employment status, 6 on full-time or part-time work status and working hours, 6 on social benefits, and 9 on independent living (Fig. 1). The average quality score on the Newcastle-Ottawa Scale was 7 (range: 6–8), indicating generally good quality and low risk of bias (Supplemental Table S1).

**Table 1:** Summary of the 11 cohorts included in the individual participant data meta-analysis.

										Adulthood socioeconomic outcomes assessed						
Cohort	Country	Birth year	Age	VP (n)	TB (n)	TB recruited	VP Male (%)	TB Male (%)	MEL	Educational attainment	Economically active	Employment status	Work status	Working hours	Social benefits	Independent living
1. AYLS	Finland	1985–86	26	32	351	Birth	56.3	45.0	2.29	**×**	**×**	**×**	**×**	**×**		**×**
2. BLS	Germany	1985–86	26	260	229	Birth	53.1	46.7	1.76	**×**	**×**	**×**	**×**	**×**	**×**	**×**
3. ESTER	Finland	1985–89	23	75	347	Birth	44.0	47.4	2.28	**×**	**×**	**×**	**×**	**×**		**×**
4. HESVA	Finland	1978–85	22	187	190	Birth	43.9	40.0	2.08	**×**	**×**	**×**				**×**
5. NTNU LBW Life	Norway	1986–88	26	50	81	Birth	50.0	46.9	2.40	**×**	**×**	**×**			**×**	**×**
6. POPS^a^	Netherlands	1983	28	317	NA	NA	38.5	NA	1.83	**×**	**×**	**×**	**×**	**×**	**×**	**×**
7. EPICure	UK + Ireland	1995	19	126	62	Childhood	46.0	38.7	1.94	**×**	**×**	**×**	**×**	**×**	**×**	**×**
8. NZVLBW	New Zealand	1986	28	250	99	Adulthood	42.8	36.0	2.10	**×**	**×**		**×**	**×**		**×**
9. VICS	Australia	1991–92	18	199	150	Adulthood	45.7	42.0	2.00	**×**	**×**	**×**			**×**	**×**
10. RWH	Australia	1977–82	26	95	22	Birth	44.2	59.1	1.46	**×**	**×**	**×**			**×**	
11. UCLH	UK	1979–84	30	104	89	Adulthood	60.6	47.2	2.41	**×**	**×**	**×**				

VP = very preterm/very low birth weight (VPT/VLBW); TB = Term-born controls. MEL = Maternal educational level, measured at birth or childhood, harmonised according to the International Standard Classification of Education (1 = Lower secondary or less; 2 = Upper secondary or short post-secondary; 3 = University education or equivalent). AYLS = Arvo Ylppo Longitudinal Study; BLS = Bavarian Longitudinal Study; ESTER = Early Life Programming of Adult Health and Disease Study; HESVA = Helsinki Study of Very Low Birth Weight Adults; NTNU LBW Life = Norwegian University of Science and Technology Low Birth Weight in a Lifetime Perspective study.

^a^POPS = Project on Premature and Small for Gestational Age Infants (only included VPT/VLBW individuals); EPICure = Extremely Preterm Birth in the UK and Ireland; NZVLBW = New Zealand Very Low Birthweight; VICS = Victorian Infant Collaborative Study; RWH = Royal Women’s Hospital; UCLH = University College London Hospitals.

Table 2 presents descriptive statistics and group comparisons for demographic and socioeconomic characteristics. Compared with the term-born peers, the VPT/VLBW group had a lower proportion of individuals of white ethnicity (89% vs. 95.4%) and mothers with university education (19.2% vs. 36.9%). They had lower average gestational age (28.6 vs. 39.8 weeks), lower birth weight (1122 vs. 3534 grams), and higher rates of multiple births (24.5% vs. 1.3%) and NSI (13.8% vs. 0.9%). Socioeconomically, the VPT/VLBW group had lower university education (14.3% vs. 25.1%), higher unemployment (17.5% vs. 12.7%), received social benefits more often (18.9% vs. 7.1%) and fewer lived independently (49.8% vs. 61.1%) than their term-born counterparts. Cohort-specific data are detailed in Supplementary Table S2.

**Table 2:** Descriptive statistics across VPT/VLBW and term-born groups (pooled data from 10 cohorts^a^).

	n (%) / M(SD)^b^			
	VPT/VLBW (*n* = 1378)	Term-borns (*n *= 1620)	χ^2^/*F*^b^	*p*
**Mean age at assessment (years)**^**b**^	24.75 (4.09)	24.63 (3.31)	0.67	0.415
**Sex**			3.02	0.082
Male	657 (47.7)	721 (44.5)		
Female	721 (52.3)	899 (55.5)		
**Ethnicity**			27.04	<0.001
White	964 (89%)	868 (95.4%)		
Non-white	119 (11%)	42 (4.6%)		
**Maternal education**^c^			121.77	<0.001
Low (ISCED 0–2)	346 (28.4)	245 (16.3)		
Middle (ISCED 3–5)	638 (52.4)	701 (46.8)		
High (ISCED 6–8)	234 (19.2)	553 (36.9)		
**Gestational age (weeks)**^b^	28.64 (2.8)	39.81 (1.2)	19121	<0.001
**Birth weight (grams)**^b^	1122 (318)	3534 (478)	24535	<0.001
**Birth weight (z score)**^b^	−0.33 (1.09)	0.01 (0.91)	81.92	<0.001
**Head circumference (cm)**	26.16 (2.56)	35.15 (1.40)	11831	<0.001
**Multiple birth**			327.19	<0.001
Singleton	1037 (75.5)	1352 (98.7)		
Multiple	336 (24.5)	18 (1.3)		
**Neurosensory impairments**			193.38	<0.001
No	1188 (86.2)	1605 (99.1)		
Yes	216 (13.8)	15 (0.9)		
**Educational attainment**^c^			87.51	<0.001
Low (ISCED 0–2)	222 (16.4)	129 (8.0)		
Middle (ISCED 3–5)	938 (69.3)	1080 (66.9)		
High (ISCED 6–8)	193 (14.3)	405 (25.1)		
**Economically active**			2.49	0.114
No	632 (47.7)	813 (50.6)		
Yes	694 (52.3)	794 (49.4)		
**Employment status**			11.96	0.003
In paid work	475 (44.1)	687 (45.5)		
*Part-time*	*110* (*24.2)*	*201* (*30.7)*	*0.69*	*0.406*
*Full-time*	*345* (*75.8)*	*454* (*69.3)*		
*Working hours*^b^	*38.84* (*10.4)*	*38.16* (*7.8)*	*1.26*	*0.263*
In education	413 (38.4)	630 (41.8)		
Unemployed	188 (17.5)	191 (12.7)		
**Receipts of social benefits**			55.91	<0.001
No	540 (77.1)	497 (92.9)		
Yes	160 (22.9)	38 (7.1)		
**Independent living**			33.29	<0.001
No	579 (50.2)	577 (38.9)		
Yes	575 (49.8)	905 (61.1)		

Italicized values indicate subcategories of employment status under "In paid work."

^a^The POPS cohort was excluded in the group comparison analysis due to the lack of term-born control group.

^b^The descriptive statistics were mean and standard deviation, coefficients for group compositions were *F* tests.

^c^Maternal education and participants’ educational attainment were harmonised based on the International Standard Classification of Education: “ISCED 0–2” = Low secondary or less; “ISCED 3–5” = Upper secondary or short post-secondary; “ISCED 6–8” = University education or equivalent.

### Differences in socioeconomic outcomes between VPT/VLBW and term-born

Table 3 presents the one-stage IPD meta-analysis results on VPT/VLBW status and adulthood socioeconomic outcomes using the random-intercept and random-slope models. Based on the model comparisons, educational attainment and independent living were better fit by random-slope models, while all other outcomes are reported using random-intercept models as the primary results. Compared with the term-born peers, VPT/VLBW adults had significantly lower odds of achieving higher educational attainment (OR = 0.40 [0.26–0.59], *d* = −0.51), remaining in education (OR = 0.63 [0.47–0.84], *d* = −0.26), or being in paid work (OR = 0.76 [0.59–0.97], *d* = −0.15), and they were more likely to receive social benefits (OR = 3.93 [2.63–5.86], *d* = 0.76). Adjusting for age, sex, and maternal education had minimal impact on the results, except that the association between VPT/VLBW status and in paid work (versus unemployment) became non-significant. Among participants without NSI, VPT/VLBW adults still showed lower odds of higher educational attainment (OR = 0.51 [0.33–0.78], *d* = −0.37) and higher odds of receiving social benefits (OR = 2.80 [1.61–4.89], *d* = 0.57), though effect sizes were slightly attenuated.

**Table 3:** One-stage IPD meta-analyses of the association between VPT/VLBW and adulthood socioeconomic outcomes (pooled data from 10 cohorts^a^).

	Cohorts (n)		Random-intercept model		Random-slope model			LRT (*p*)
Outcomes		N	OR [95%CI]	*p*	OR [95%CI]	*p*	*τ*^2^	
**Educational attainment**	10							
Unadjusted model		2967	**0.47 [0.40, 0.56]**	**<0.001**	**0.40 [0.26, 0.59]**	**<0.001**	0.32	<0.001
Adjusted for age, sex, maternal education		2695	**0.52 [0.44, 0.61]**	**<0.001**	**0.44 [0.28, 0.69]**	**<0.001**	0.40	<0.001
Excluding participants with NSI		2525	**0.59 [0.50, 0.71]**	**<0.001**	**0.51 [0.33, 0.78]**	**0.002**	0.36	<0.001
**Economically active**	10							
Unadjusted model		2933	0.92 [0.77, 1.10]	0.344	0.92 [0.77, 1.10]	0.364	0.00	0.992
Adjusted for age, sex, maternal education		2655	0.86 [0.71, 1.04]	0.121	0.85 [0.68, 1.05]	0.138	0.02	0.862
Excluding participants with NSI		2477	0.94 [0.77, 1.15]	0.576	0.93 [0.73, 1.18]	0.551	0.03	0.838
**Employment status**^b^	9							
***In education (ref. Unemployed)***								
Unadjusted model		2584	**0.63 [0.47, 0.84]**	**0.002**	**0.51 [0.36, 0.74]**	**<0.001**	0.07	0.383
Adjusted for age, sex, maternal education		2314	**0.72 [0.53, 0.98]**	**0.038**	**0.61 [0.42, 0.89]**	**0.011**	0.07	0.694
Excluding participants with NSI		2156	0.88 [0.63, 1.24]	0.469	0.74 [0.50, 1.09]	0.127	0.04	0.789
***In paid work (ref. Unemployed)***								
Unadjusted model		2584	**0.76 [0.59, 0.97]**	**0.031**	**0.60 [0.43, 0.83]**	**0.002**	0.07	0.383
Adjusted for age, sex, maternal education		2314	0.78 [0.59, 1.01]	0.064	**0.65 [0.46, 0.91]**	**0.012**	0.07	0.694
Excluding participants with NSI		2156	0.94 [0.71, 1.26]	0.697	0.79 [0.55, 1.13]	0.194	0.07	0.789
**Work status (full vs. part-time)**	5							
Unadjusted model		901	1.04 [0.68, 1.59]	0.852	0.96 [0.53, 1.73]	0.890	0.13	0.584
Adjusted for age, sex, maternal education		891	0.89 [0.57, 1.41]	0.632	0.83 [0.44, 1.60]	0.584	0.18	0.630
Excluding participants with NSI		843	1.03 [0.63, 1.68]	0.902	1.00 [0.53, 1.89]	0.994	0.11	0.767
**Working hours per week [B**^c^**]**	5							
Unadjusted model		900	0.58 [−0.77, 1.90]	0.397	−0.28 [−4.34, 2.67]	0.853	5.60	0.279
Adjusted for age, sex, maternal education		890	−0.05 [−1.48, 1.14]	0.938	−0.39 [−2.16, 1.12]	0.783	1.89	0.944
Excluding participants with NSI		842	0.43 [−0.91, 1.66]	0.516	0.37 [−1.68, 1.69]	0.602	0.23	0.787
**Social benefits**	5							
Unadjusted model		1235	**3.93 [2.63, 5.86]**	**<0.001**	**8.97 [2.67, 29.83]**	**<0.001**	0.82	0.056
Adjusted for age, sex, maternal education		1032	**3.98 [2.37, 6.69]**	**<0.001**	**7.71 [2.34, 25.34]**	**<0.001**	0.77	0.055
Excluding participants with NSI		920	**2.80 [1.61, 4.89]**	**<0.001**	**4.94 [1.59, 15.34]**	**0.006**	0.54	0.206
**Independent living**	8							
Unadjusted model		2636	**0.63 [0.51, 0.77]**	**<0.001**	0.67 [0.41, 1.09]	0.105	0.36	<0.001
Adjusted for age, sex, maternal education		2415	**0.66 [0.53, 0.82]**	**<0.001**	0.72 [0.40, 1.27]	0.254	0.50	<0.001
Excluding participants with NSI		2249	0.84 [0.67, 1.07]	0.155	0.81 [0.45, 1.46]	0.490	0.49	<0.001

Bolded coefficients indicate statistically significant at the *p* < 0.05 level and their corresponding confidence intervals. LRT = Likelihood Ratio Test. A significant *p* value from the LRT indicates that the random-slope model provides a significantly better fit than the random-intercept model.

^a^The Project on Preterm and Small-for-Gestational-Age Infants (POPS) cohort was excluded in the group comparison analysis due to the lack of term-born control group. Supplementary Table S3 showed the results of sensitivity analysis that included POPS.

^b^Employment status was an unordered categorical variable, coded as 0 = unemployed, 1 = in education/training; 2 = in paid work.

^c^B indicates the unstandardised linear regression coefficient. NSI = neurosensory impairments.

For independent living, the random-intercept model showed that VPT/VLBW adults had significantly lower odds of living independently (OR = 0.63 [0.51–0.77], *d* = −0.26), while this association was not significant in the random-slope model (OR = 0.67 [0.41–1.09], *d* = −0.22). Further cohort-level analysis showed that among the eight available cohorts, only BLS, HESVA, and EPICURE showed significantly lower odds for VPT/VLBW adults, while ESTER showed higher odds, and the remaining four cohorts showed no significant associations (Table S5). There were no significant associations between VPT/VLBW status and being economically active, full-time or part-time employed, or weekly working hours among those employed.

### Predictors of socioeconomic outcomes within VPT/VLBW adults

Table 4 presents univariable and multivariable IPD analyses of predictors on educational attainment, employment status, receipt of social benefits, and independent living among VPT/VLBW individuals. The text below reports results from the multivariable analyses as primary findings, with predictors presented in order of effect size, from largest to smallest. Results for economically active, full-time or part-time work status, and weekly work hours are available in Supplementary Table S6.

**Table 4:** Univariable and multivariable regressions of predictors on socioeconomic outcomes among VPT/VLBW individuals.

	Educational attainment (*n* = 1638)			
	univariable		multivariable	
	OR [95%CI]	*p*	OR [95%CI]	*p*
Age (per year)	**1.12 [1.09, 1.15]**	<0.001^***^	**1.10 [1.07, 1.14]**	<0.001^***^
Sex (Female)	**1.52 [1.24, 1.85]**	<0.001^***^	**1.56 [1.26, 1.92]**	<0.001^***^
Gestational age (per week)	**1.13 [1.09, 1.17]**	<0.001^***^	**1.11 [1.01, 1.23]**	0.037^*^
Birthweight z score (per SD)	1.02 [0.94, 1.11]	0.687	**1.32 [1.13, 1.54]**	<0.001^***^
Head circumference (per cm)	**1.13 [1.08, 1.18]**	<0.001^***^	0.96 [0.88, 1.05]	0.352
Multiple birth	1.04 [0.82, 1.31]	0.746	1.06 [0.83, 1.35]	0.643
IVH	**0.66 [0.53, 0.84]**	<0.001^***^	0.85 [0.67, 1.09]	0.207
BPD	**0.38 [0.30, 0.48]**	<0.001^***^	**0.52 [0.40, 0.67]**	<0.001^***^
NSI	**0.27 [0.20, 0.37]**	<0.001^***^	**0.32 [0.23, 0.44]**	<0.001^***^
Higher maternal education	**1.92 [1.65, 2.24]**	<0.001^***^	**1.89 [1.61, 2.22]**	<0.001^***^
Maternal age at birth (per year)	**1.02 [1.01, 1.04]**	0.039^*^	**1.02 [1.00, 1.04]**	0.033^*^
Ethnicity (non-white)	**0.68 [0.48, 0.96]**	0.027^*^	0.80 [0.56, 1.16]	0.237

	Employment status (*n* = 1390)							
	In education (reference: Unemployed)				In paid work (reference: Unemployed)			
	univariable		multivariable		univariable		multivariable	
	OR [95%CI]	*p*	OR [95%CI]	*p*	OR [95%CI]	*p*	OR [95%CI]	*p*
Age (per year)	**0.75 [0.66, 0.85]**	<0.001^***^	**0.73 [0.65, 0.83]**	<0.001^***^	**1.12 [1.03, 1.21]**	0.005^**^	**1.11 [1.03, 1.20]**	0.008^**^
Sex (Female)	1.13 [0.78, 1.65]	0.518	1.03 [0.69, 1.53]	0.893	0.80 [0.58, 1.10]	0.162	0.75 [0.53, 1.04]	0.088
Gestational age (per week)	1.08 [0.99, 1.18]	0.093	1.19 [0.98, 1.44]	0.082	**1.07 [1.00, 1.15]**	0.048^*^	1.09 [0.92, 1.28]	0.311
Birthweight z score (per SD)	0.98 [0.82, 1.16]	0.803	1.29 [0.96, 1.74]	0.087	1.00 [0.87, 1.14]	0.965	1.15 [0.89, 1.47]	0.281
Head circumference (per cm)	1.02 [0.98, 1.05]	0.753	0.87 [0.74, 1.02]	0.086	1.06 [0.98, 1.15]	0.140	0.96 [0.84, 1.09]	0.522
Multiple birth	1.21 [0.78, 1.88]	0.387	1.19 [0.76, 1.88]	0.445	0.91 [0.62, 1.32]	0.608	0.91 [0.62, 1.34]	0.629
IVH	**0.54 [0.34, 0.86]**	0.009^**^	**0.59 [0.36, 0.98]**	0.043^*^	**0.62 [0.42, 0.90]**	0.013^*^	0.72 [0.48, 1.07]	0.102
BPD	0.71 [0.46, 1.10]	0.121	0.86 [0.52, 1.41]	0.544	**0.67 [0.46, 0.97]**	0.035^*^	0.80 [0.53, 1.20]	0.286
NSI	**0.33 [0.21, 0.54]**	<0.001^***^	**0.39 [0.24, 0.65]**	<0.001^***^	**0.33 [0.22, 0.50]**	<0.001^***^	**0.35 [0.23, 0.52]**	<0.001^***^
Higher maternal education	**1.48 [1.07, 2.06]**	0.018^*^	1.29 [0.90, 1.84]	0.166	0.79 [0.60, 1.02]	0.069	**0.73 [0.56, 0.95]**	0.020^*^
Maternal age at birth (per year)	**1.07 [1.03, 1.11]**	<0.001^***^	**1.06 [1.02, 1.11]**	0.005^**^	1.01 [0.98, 1.05]	0.513	1.02 [0.98, 1.06]	0.337
Ethnicity (non-white)	0.84 [0.38, 1.83]	0.662	0.83 [0.35, 1.97]	0.680	0.67 [0.32, 1.39]	0.285	0.75 [0.35, 1.60]	0.456

	Social benefits (*n* = 1014)				Independent living (*n* = 1468)			
	univariable		multivariable		univariable		multivariable	
	OR [95%CI]	*p*	OR [95%CI]	*p*	OR [95%CI]	*p*	OR [95%CI]	*p*
Age (per year)	1.08 [0.77, 1.52]	0.644	1.01 [0.81, 1.26]	0.906	**1.31 [1.15, 1.48]**	<0.001^***^	**1.41 [1.26, 1.58]**	<0.001^***^
Sex (Female)	0.82 [0.58, 1.15]	0.248	0.98 [0.67, 1.43]	0.914	**2.01 [1.55, 2.59]**	<0.001^***^	**2.11 [1.59, 2.80]**	<0.001^***^
Gestational age (per week)	**0.87 [0.79, 0.95]**	0.002^**^	0.83 [0.68, 1.02]	0.071	1.05 [0.99, 1.11]	0.103	1.03 [0.89, 1.19]	0.712
Birthweight z score (per SD)	1.02 [0.88, 1.19]	0.763	0.75 [0.56, 1.02]	0.066	1.01 [0.91, 1.12]	0.862	1.18 [0.95, 1.47]	0.144
Head circumference (per cm)	0.91 [0.82, 1.00]	0.056	1.04 [0.88, 1.22]	0.655	1.04 [0.98, 1.11]	0.173	0.99 [0.88, 1.12]	0.872
Multiple birth	1.34 [0.92, 1.95]	0.129	1.41 [0.93, 2.14]	0.107	1.22 [0.91, 1.65]	0.187	1.15 [0.83, 1.60]	0.398
IVH	**1.97 [1.35, 2.88]**	<0.001^***^	**1.66 [1.07, 2.59]**	0.026^*^	**0.66 [0.48, 0.91]**	0.010^*^	0.72 [0.51, 1.03]	0.076
BPD	**1.50 [1.02, 2.22]**	0.042^*^	1.11 [0.70, 1.77]	0.649	**0.63 [0.47, 0.85]**	0.003^**^	0.80 [0.57, 1.12]	0.192
NSI	**7.72 [4.85, 12.28]**	<0.001^***^	**6.68 [4.11, 10.86]**	<0.001^***^	**0.20 [0.14, 0.30]**	<0.001^***^	**0.20 [0.13, 0.30]**	<0.001^***^
Higher maternal education	**0.71 [0.53, 0.96]**	0.028^*^	0.79 [0.54, 1.16]	0.247	**1.32 [1.09, 1.61]**	0.005^**^	**1.39 [1.12, 1.72]**	0.003^**^
Maternal age at birth (per year)	0.98 [0.95, 1.01]	0.153	0.98 [0.95, 1.02]	0.307	**0.97 [0.94, 0.99]**	0.008^**^	**0.96 [0.93, 0.98]**	0.002^**^
Ethnicity (non-white)	1.56 [0.88, 2.78]	0.129	1.31 [0.68, 2.51]	0.416	**0.46 [0.27, 0.80]**	0.006^**^	**0.39 [0.21, 0.70]**	0.002^**^

Bolded coefficients indicate statistically significant at the *p* < 0.05 level and corresponding confidence intervals. IVH = intraventricular haemorrhage, BPD = bronchopulmonary dysplasia, NSI = neurosensory impairments. ^*^p < 0.05, ^**^p < 0.01, ^***^p < 0.001.

#### Educational attainment

The presence of NSI (OR = 0.32 [0.23–0.44], *d* = −0.63) and BPD (OR = 0.52 [0.40–0.67], *d* = −0.36) were both significantly associated with lower educational attainment. Conversely, higher maternal education (OR = 1.89 [1.61–2.22], *d* = 0.35), female sex (OR = 1.56 [1.26–1.92], *d* = 0.25), higher birthweight z-score (OR_per SD_ = 1.32 [1.13–1.54], *d* = −0.15), higher gestational age (OR_per week_ = 1.11 [1.01–1.23], *d* = 0.06), older age at assessment (OR_per year_ = 1.10 [1.07–1.14], *d* = 0.05), and higher maternal age at birth (OR _per year_ = 1.02 [1.00–1.04], *d* = 0.01) were significantly associated with higher educational attainment.

#### Employment status

NSI significantly reduced the odds of being in education (OR = 0.39 [0.24–0.65], *d* = −0.52) and in paid work (OR = 0.35 [0.23–0.52], *d* = −0.58). IVH also lowered the odds of being in education (OR = 0.59 [0.36–0.98], *d* = −0.29). Oolder age at assessment was associated with lower odds of remaining in education (OR_per year_ = 0.73 [0.65–0.83], *d* = −0.17) but higher odds of being in paid work (OR_per year_ = 1.11 [1.03–1.20], *d* = 0.06). Higher maternal age at birth was also associated with higher odds of being in education (OR_per year_ = 1.06 [1.02–1.11], *d* = 0.03).

#### Social benefits

NSI (OR = 6.68 [4.11–10.86], *d* = 1.05) and IVH (OR = 1.66 [1.07–2.59], *d* = 0.28) were both associated with higher odds of receiving social benefits in the multivariable model.

#### Independent living

NSI (OR = 0.2 [0.13–0.30], *d* = −0.89), non-white ethnicity (OR = 0.39 [0.21–0.70], *d* = −0.52) and older maternal age at birth (OR_per year_ = 0.96 [0.93–0.98], *d* = −0.02) were associated with lower odds of living independently. In contrast, female sex (OR = 2.11 [1.59–2.80], *d* = 0.41) older age at assessment (OR_per year_ = 1.41 [1.26–1.58], *d* = 0.19), and higher maternal education (OR = 1.39 [1.12–1.72], *d* = 0.18) were associated with higher odds of living independently.

## Discussion

Using IPD from 11 cohorts across eight countries in Europe, Australia and New Zealand, we found that VPT/VLBW birth was associated with lower odds of achieving higher educational attainment, being in paid work or remaining in education, as well as higher odds of receiving social benefits during emerging adulthood (18–30 years). These disparities remained significant after adjusting for age, sex, and maternal education. Excluding participants with NSI reduced group differences, but disadvantages in educational attainment and receipt of social benefits for VPT/VLBW persisted. Among VPT/VLBW adults, NSI was a major risk factor for all socioeconomic outcomes, with the strongest association observed for receiving social benefits. Higher gestational age and birth weight were associated with better educational attainment. Maternal education was positively associated with educational attainment, remaining in education, and living independently. Maternal age was positively associated with higher educational attainment but negatively associated with independent living. Non-white ethnicity, male sex, and younger age were also associated with lower odds of living independently.

### Comparing group differences with previous studies

Aligned with a previous aggregate meta-analysis,^14^ this IPD meta-analysis found that VPT/VLBW adults were less likely to achieve higher educational attainment (small-to medium-effect size), be in paid work or education (small effect sizes), and more likely to receive social benefits (medium-to-large effect size), with slightly larger effect sizes observed here. These findings also align with recent population-based registry studies (which could not be included in the IPD due to data restriction), including a Canadian study^48^ and two Danish VPT/VBLW studies,^11,19^ which similarly reported educational and economic disadvantages among VPT/VLBW adults compared to their term-born or normal-birth-weight peers. These results reinforce the long-term socioeconomic challenges faced by VPT/VLBW populations across high-income countries. Adjusting for age, sex, and maternal education, did not alter group differences across outcomes. However, when participants with NSI were excluded, differences persisted only for educational attainment and receipt of social benefits. These findings suggest that while NSI significantly contributes to socioeconomic disparities, it is not the sole driver, highlighting the need for further research to uncover other risk factors.

Notably, a subgroup analysis from a prior aggregate meta-analysis^14^ comparing cohort and registry studies found that, based on five cohort studies, individuals born preterm or with low birth weight had lower odds of living independently compared with term-borns. However, even with the standardised harmonisation of individual-level data, our findings still showed mixed results regarding the association between VPT/VLBW status and independent living. Cohort-level analyses indicated considerable variability, even within the same country. For example, among the three Finnish cohorts, AYLS showed no significant association, HESVA showed a negative association, while ESTER showed a positive association. These discrepancies may be influenced by differences in social support networks, education, and healthcare systems across countries and regions—factors that were not fully accounted for in the current IPD analyses. Additionally, cohort-level variations in birth years, participant age at assessment, and sample sizes may also contribute to these inconsistencies. Future studies should explore the role of policy frameworks, access to support services, and individual life-course transitions in independent living outcomes.

Given ongoing improvements in medical care,^3^ there might be concerns about the generalisability of findings from earlier cohorts to more recent generations. However, studies comparing cohorts born before and after the millennium show no significant improvements in neurodevelopmental outcomes,^49–51^ and even slight declines in behavioural outcomes^52^ and health-related quality of life.^53^ This suggests that increased survival has not yet translated into improved psychosocial or socioeconomic outcomes. Therefore, our findings remain relevant for current generations of VPT/VLBW individuals until newer cohort data are available. As a growing number of VPT/VLBW infants survive to adulthood, more will face socioeconomic challenges.^4,54^ Future research may focus on identifying protective factors and effective interventions to mitigate these challenges and narrow the socioeconomic achievement gaps between VPT/VLBW and term-born individuals.

### Factors associated with VPT/VLBW’s socioeconomic outcomes

NSI was strongly associated with all these socioeconomic outcomes, with medium-to-large effect sizes for educational attainment and employment status, and large effects for receiving social benefits and independent living. Supporting this, a Swedish national cohort study found that VPT individuals were nearly four times more likely to require economic assistance due to disability or persistent illness compared to term-born peers, even after adjusting for socioeconomic and perinatal factors.^12^ These findings highlight the critical role of disability and developmental impairments in driving long-term socioeconomic challenges for VPT/VLBW individuals. Preventing NSI and mitigating its impact should remain a central focus of neonatal care advancements.^10,18,55,56^

For factors associated with educational attainment, higher maternal education had a small-to-medium effect, while higher gestational age and birth weight showed small effects. This result highlights the enduring impact of socioeconomic conditions in the family of origin on educational paths, along with the additive effects of VPT and/or VLBW. Similar findings were reported in four Nordic countries, where lower parental education exacerbated the educational challenges of being born preterm.^34^ For instance, the relative risk of low educational attainment was 1.84 for Danish VPT adults whose parents had high educational levels, and 5.25 for those whose parents had low educational levels. Despite the protective effect of higher parental education, it did not mitigate the educational challenges associated with being born preterm. Besides, lower maternal education was associated with higher odds of being in paid work but reduced odds of pursuing further education or living independently, suggesting that individuals from less advantaged backgrounds may prioritise workforce entry over continued education. A recent Canadian population-based matched cohort study reported that preterm birth was associated with lower adulthood income, lower upward social mobility, and higher downward mobility, particularly among individuals from economically disadvantaged families.^57^ These results emphasise the need for targeted interventions that consider family socioeconomic backgrounds.

Older maternal age at birth was positively associated with higher educational attainment in VPT/VLBW offspring, mirroring trends observed in the general population where children of older mothers may benefit from higher maternal education, more stable socioeconomic conditions, greater parental experience, and enriched home environments.^58–60^ However, VPT/VLBW adults born to older mothers were less likely to live independently, even after controlling for NSI, potentially due to additional health or developmental challenges requiring extended familial support.^61,62^ Alternatively, older mothers may have greater financial stability and better housing conditions, allowing their children to remain at home longer. This trend also aligns with modern societal patterns, where emerging adults, particularly those who are unmarried and with limited economic resources, increasingly reside with their parents.^63^ Non-white VPT/VLBW adults were also less likely to live independently, potentially reflecting cultural norms emphasising collectivism and family cohesion or practical barriers, such as difficulties securing independent housing in their twenties. Additionally, older age and female sex were positively associated with higher educational attainment and independent living, consistent with patterns observed in the general population.^64,65^

It is worth noting that VPT/VLBW birth itself is also influenced by social determinants of health, including socioeconomic status, racial and ethnic disparities, environmental exposures, and access to quality prenatal care.^66^ Structural inequalities—such as barriers to healthcare, financial instability, and neighbourhood-level deprivation—can contribute to higher rates of preterm birth,^67^ perpetuating a cycle of disadvantage into adulthood. Maternal age and education, included as covariates in our analyses, may serve as proxies for broader socioeconomic conditions that shape both perinatal health and long-term opportunities. Additionally, medically complex deliveries, which are more common in preterm infants, may further widen economic disparities by increasing neonatal healthcare costs, long-term medical expenses, and caregiving responsibilities for families.^68^ Given these complex interrelations, future research should explore how multilevel socioeconomic factors interact with biological vulnerabilities to shape long-term trajectories, and how targeted policy interventions can mitigate these disparities.

Beyond these factors, other covariates such as cognitive and academic achievement play a key role in socioeconomic outcomes. A meta-analysis showed that preterm or low-birth-weight individuals perform worse in mathematics compared with their term-born peers, particularly those born before 28 weeks’ gestation.^69^ An IPD meta-analysis reported that per standard deviation increase in mathematics z-score is associated with a 1.36-fold increase in the odds of attending postsecondary education.^70^ Moreover, the link between preterm birth and lower wealth at age 42 was mediated by lower cognitive abilities, reading skills, mathematical performance in middle childhood, as well as lower educational qualifications in young adulthood.^22^ Therefore, other factors including mathematical skills,^22,69,70^ school grades and special education attendance,^21^ cognitive abilities,^5,13,71^ and behavioural and peer problems^17,72^ may serve as mediators between VPT/VLBW status and socioeconomic underachievement and as potential intervention targets. Future studies should explore mediation models within an IPD framework to better understand how these pathways operate in life course models and to inform targeted interventions.

### Limitations and future directions

Several limitations should be acknowledged. First, inclusion criteria for gestational age and birth weight varied across cohorts. While most cohorts included VPT (<32 weeks) or VLBW (<1500 g) samples, the EPICure study restricted inclusion to extremely preterm individuals born at 22–26 weeks’ gestation.^73^ Second, recruitment strategies for term-born control groups differed by timing (birth, childhood, or adulthood) and criteria (e.g., sociodemographic-matched, geographically defined, or school-based). Although most cohorts recruited controls at birth from the same region, others, like the NZVLBW cohort, used peer nominations or random sampling in adulthood, while the EPICure cohort selected classroom peers at age 6 through mainstream schools. These variations may introduce heterogeneity across studies. Third, the focus on emerging adulthood (18–30 years)^74^ limits generalisability to later life stages. Further studies should examine whether VPT/VLBW individuals catch up in socioeconomic achievements or face persistent disadvantages into established adulthood (30–45 years), a period often associated with increased economic responsibilities, such as raising children. Fourth, we did not systematically search for cohorts outside the RECAP and APIC consortia, which may have led to the exclusion of other relevant datasets. Lastly, all cohorts were from high-income countries, where institutional and financial support for VPT/VLBW individuals is more accessible.^75^ Yet, socioeconomic disparities persist in these settings. The lack of studies from low- and middle-income countries highlights a gap in the global understanding of VPT/VLBW adult socioeconomic outcomes.

## Conclusions

Most VPT/VLBW individuals transition into adulthood similarly to their term-born peers. Differences in educational attainment, participation in paid work or education, and receipt of social benefits are largely driven by the high prevalence of NSI within this population. However, even among those without NSI, VPT/VLBW adults still show lower educational attainment and higher rates of receiving social benefits. Additional risk factors that include lower gestational age, lower birth weight, medical complications, lower maternal education, younger maternal age, and non-white ethnicity, reflect the sociodemographic profile of preterm birth. Despite rising survival in recent decades, there has been no corresponding improvements in cognitive, social, or behavioural outcomes. Consequently, increasing numbers of VPT/VLBW individuals may require targeted support, particularly those from socioeconomically disadvantaged families. Greater efforts are needed to reduce the prevalence of NSI and enhance educational support during childhood for this population, such as improved training for educators to better understand and address the specific needs of preterm-born children and adults.^76,77^

## Supplementary information

IPD CONSORT checklist

Supplementary

Table S2

## Data Availability

Information regarding the data availability can be found at: https://recap-preterm.eu/for-scientists/the-recap-preterm-cohort-platform/.
