# Supplementary material for: Socioeconomic outcomes in very preterm/very low birth weight adults: individual participant data meta-analysis
Source: Pediatr Res. 2025 May 3;98(6):2401–13. doi: 10.1038/s41390-025-04082-1 (PMC12811119; doi:10.1038/s41390-025-04082-1)
Supplement: Supplementary file 2 — Supplementary [file 41390_2025_4082_MOESM2_ESM.pdf]

# Socioeconomic outcomes in very preterm/very low birth weight adults: Individual participant data meta-analysis

## Supplementary Information

**Table S1.** Risk of bias assessment using the Newcastle-Ottawa quality assessment form for cohort studies

| Cohort | Selection | | | | Comparability | Outcome | | | Total score | Quality |
|---|---|---|---|---|---|---|---|---|---|---|
| | Representativeness of the exposed cohort | Selection of the non-exposed cohort | Ascertainment of exposure | Outcome of interest was not present at start of study | Comparable on design or controlled for confounders | Assessment of outcome | Follow-up long enough | Adequacy follow up | | |
| AYLS | Truly★ | Same community★ | Secure record★ | Yes★ | ★★ | Self-report | Yes★ | follow-up rate < 50% | 7 | good |
| BLS | Truly★ | Same community★ | Secure record★ | Yes★ | ★★ | Self-report | Yes★ | follow-up rate < 50% | 7 | good |
| ESTER | Truly★ | Same community★ | Secure record★ | Yes★ | ★★ | Self-report | Yes★ | >50% or no bias★ | 7 | good |
| HESVA | Truly★ | Same community★ | Secure record★ | Yes★ | ★★ | Self-report | Yes★ | follow-up rate < 50% | 7 | good |
| NTNU LBW Life | Truly★ | Different source | Secure record★ | Yes★ | ★★ | Self-report | Yes★ | >50% or no bias★ | 7 | good |
| POPS | Truly★ | NA | Secure record★ | Yes★ | ★★ | Self-report | Yes★ | >50% or no bias★ | 7 | good |
| EPICure | Truly★ | Different source | Secure record★ | Yes★ | ★★ | Self-report | Yes★ | follow-up rate < 50% | 6 | fair |
| NZVLBW | Truly★ | Different source | Secure record★ | Yes★ | ★★ | Self-report | Yes★ | >50% or no bias★ | 7 | good |
| VICS | Truly★ | Same community★ | Secure record★ | Yes★ | ★★ | Self-report | Yes★ | >50% or no bias★ | 8 | good |
| RWH | Truly★ | Different source | Secure record★ | Yes★ | ★★ | Self-report | Yes★ | >50% or no bias★ | 7 | good |
| UCLH | Truly★ | Different source | Secure record★ | Yes★ | ★★ | Self-report | Yes★ | >50% or no bias★ | 7 | good |

***Note.*** A study can be awarded a maximum of one star for each numbered item within the Selection and Outcome categories. A maximum of two stars can be given for Comparability. Each cohort could achieve a maximum score of 9, categorized as follows: Poor quality (0–3 points), Fair quality (4–6 points), and Good quality (7–9 points).[41]

**NEWCASTLE-OTTAWA QUALITY ASSESSMENT SCALE COHORT STUDIES**

**Selection**

1) Representativeness of the exposed cohort

a) truly representative of the average _VPT/VLBW_ (describe) in the community ★

b) somewhat representative of the average _VPT/VLBW_ in the community ★

c) selected group of users e.g. nurses, volunteers

d) no description of the derivation of the cohort

2) Selection of the non exposed cohort

a) drawn from the same community as the exposed cohort ★
b) drawn from a different source
c) no description of the derivation of the non exposed cohort

3) Ascertainment of exposure

a) secure record (e.g., surgical records) ★
b) structured interview ★
c) written self report
d) no description

4) Demonstration that outcome of interest was not present at start of study

a) yes ★
b) no

**Comparability**

1) Comparability of cohorts on the basis of the design or analysis

a) study controls for SES/age/sex ratios (select the most important factor) ★
b) study controls for any additional factor ★ (This criteria could be modified to indicate specific control for a second important factor.)

**Outcome**

1) Assessment of outcome

a) independent blind assessment ★
b) record linkage ★
c) self report
d) no description

2) Was follow-up long enough for outcomes to occur

a) yes (select an adequate follow up period for outcome of interest) ★
b) no

3) Adequacy of follow up of cohorts

a) complete follow up - all subjects accounted for ★
b) subjects lost to follow up unlikely to introduce bias - small number lost - > 50 % (select an adequate %) follow up, or description provided of those lost) ★
c) follow up rate < 50 % (select an adequate %) and no description of those lost
d) no statement

**Table S3.** One-stage IPD meta-analyses for the associations between VPT/VLBW and adulthood socioeconomic outcomes (all cohorts included)

| | | | **Unadjusted** | | **Adjusted[a]** | | **NSI participants excluded** | |
|---|---|---|---|---|---|---|---|---|
| | cohorts | N | OR (95%CI) | *p*-value | OR (95%CI) | *p*-value | OR (95%CI) | *p*-value |
| Educational attainment | 11 | 3253 | **0.62 (0.54, 0.72)** | **< .001*****| **0.65 (0.56, 0.76)** | **< .001***** | **0.73 (0.62, 0.86)** | **< .001***** |
| Economically active | 11 | 3251 | 0.93 (0.77, 1.11) | .400 | 0.89 (0.73, 1.08) | .253 | 0.98 (0.78, 1.24) | .879 |
| Employment status[b] | 10 | 2898 | | | | | | |
| *In education vs. Unemployed* | | | **0.64 (0.43, 0.93)** | **.002**** | **0.72 (0.52, 0.99)** | **.041*** | 0.90 (0.60, 1.35) | .537 |
| *In paid work vs. Unemployed* | | | **0.77 (0.63, 0.94)** | **.042*** | **0.79 (0.64, 0.98)** | .087 | 0.98 (0.77, 1.25) | .890 |
| Work status (full-time vs. part-time) | 6 | 1164 | 0.86 (0.47, 1.57) | .623 | 0.91 (0.57, 1.45) | .699 | 1.02 (0.48, 2.15) | .964 |
| Working hours [B] | 6 | 1163 | 0.54 (-6.26, 1.85) | .414 | 0.20 (-1.63, 1.44) | .756 | 0.67 (-1.06, 1.94) | .299 |
| Social benefits | 6 | 1549 | **5.39 (2.48, 11.73)** | **< .001***** | **5.18 (2.16, 12.49)** | **< .001***** | **3.07 (1.45, 6.48)** | **.003**** |
| Independent living | 9 | 2951 | 0.70 (0.43, 1.13) | .148 | 0.77 (0.47, 1.25) | .290 | 0.96 (0.59, 1.58) | .884 |

***Note.*** [a]adjusted for covariates including age at assessment, sex, and maternal education. [b]Employment status was coded as 0 = unemployed, 1= in education/training, 2= in paid work. $^{*}p < .05$, $^{**}p < .01$, $^{***}p < .001$.

**Table S4.** The association between VPT/VLBW and adulthood socioeconomic outcomes, excluding participants with NSI

| | **Original results** (assuming missing as non-NSI) | | | **Raw data of NSI** | | | **Multiple imputations** | | |
|---|---|---|---|---|---|---|---|---|---|
| | N | OR (95%CI) | *p* | N | OR (95%CI) | *p* | N | OR (95%CI) | *p* |
| **Educational attainment** | *2525* | **0.59 (0.50, 0.71)** | **<.001** | *2525* | **0.51 (0.33, 0.78)** | **.002** | *2967* | **0.59 (0.48, 0.71)** | **.009** |
| **Economically active** | *2477* | 0.94 (0.77, 1.15) | .576 | *2477* | 0.93 (0.73, 1.18) | .551 | *2796* | 0.96 (0.78, 1.17) | .656 |
| **Employment status** | *2156* | | | *2156* | | | *2408* | | |
| *In education vs. Unemployed* | | 0.88 (0.63, 1.24) | .469 | | 0.74 (0.50, 1.09) | .127 | | 0.81 (0.58, 1.13) | .213 |
| *In paid work vs. Unemployed* | | 0.94 (0.71, 1.26) | .697 | | 0.79 (0.55, 1.13) | .194 | | 0.89 (0.67, 1.19) | .445 |
| **Work status (full vs. part-time)** | *843* | 1.03 (0.63, 1.68) | .902 | *764* | 1.00 (0.53, 1.89) | .881 | *853* | 1.04 (0.60, 1.80) | .878 |
| **Working hours per week [B]** | *842* | 0.43 (-0.91, 1.66) | .516 | *842* | 0.43 (-1.25, 1.68) | .516 | *900* | 0.58 (-0.87, 2.04) | .430 |
| **Social benefits** | *920* | **2.80 (1.61, 4.89)** | **<.001** | *913* | **2.69 (1.53, 4.71)** | **<.001** | *1235* | **2.57 (1.62, 4.07)** | **<.001** |
| **Independent living** | *2249* | 0.84 (0.67, 1.07) | .155 | *2145* | 0.81 (0.64, 1.04) | .103 | *2636* | 0.79 (0.61, 1.03) | .085 |

***Note.*** Educational attainment and independent living were analysed using random-slope models, while random-intercept models were used for all other outcomes, due to better model fits, as shown in Table 3.

**Table S5.** Cohort-level association between VPT/VLBW and socioeconomic outcomes.

| Outcomes | Cohorts | Country | **OR** | **LCI** | **UCI** | ***p* value** |
|---|---|---|---|---|---|---|
| ***Educational attainment*** | | | | | | |
| | 1. AYLS | Finland | 0.77 | 0.38 | 1.59 | .485 |
| | **2. BLS** | **Germany** | **0.42** | **0.29** | **0.61** | **<.001** |
| | 3. ESTER | Finland | 1.13 | 0.6 | 2.15 | .708 |
| | 4. HESVA | Finland | 0.67 | 0.42 | 1.08 | .099 |
| | **5. NTNU LBW Life** | **Norway** | **0.29** | **0.14** | **0.59** | **.001** |
| | **7. EPICure** | **UK + Ireland** | **0.48** | **0.3** | **0.76** | **.002** |
| | 8. NZVLBW | New Zealand | 0.56 | 0.28 | 1.13 | .106 |
| | **9. VICS** | **Australia** | **0.09** | **0.03** | **0.29** | **<.001** |
| | **10. RWH** | **Australia** | **0.26** | **0.14** | **0.47** | **<.001** |
| | 11. UCLH | UK | 0.77 | 0.38 | 1.59 | .485 |
| ***Social benefits*** | | | | | | |
| | **2. BLS** | **Germany** | **9.3** | **1.18** | **73.23** | **.034** |
| | **5. NTNU LBW life** | **Norway** | **28.10** | **1.57** | **503.81** | **<.001** |
| | **7. EPICURE** | **UK + Ireland** | **10.74** | **3.17** | **36.45** | **<.001** |
| | **9. VICS** | **Australia** | **2.40** | **1.49** | **3.92** | **<.001** |
| | 10.RWH | Australia | 6.86 | 0.87 | 53.86 | .067 |
| ***Independent living*** | | | | | | |
| | 1. AYLS | Finland | 0.82 | 0.23 | 2.86 | .754 |
| | **2. BLS** | **Germany** | **0.39** | **0.27** | **0.58** | **<.001** |
| | **3. ESTER** | **Finland** | **1.89** | **1.13** | **3.18** | **.016** |
| | **4. HESVA** | **Finland** | **0.65** | **0.42** | **1.00** | **.049** |
| | 5. NTNU LBW life | Norway | 0.57 | 0.14 | 2.38 | .437 |
| | **7. EPICURE** | **UK + Ireland** | **0.20** | **0.10** | **0.41** | **<.001** |
| | 8. NZVLBW | New Zealand | 0.78 | 0.42 | 1.45 | .435 |
| | 9. VICS | Australia | 1.42 | 0.55 | 3.64 | .471 |

**Table S6.** One-stage IPD analysis of predictors for other socioeconomic outcomes within VPT/VLBW population

| | **Economically active** (n=1643) | | | | **Either in education, employment, or training** (n=1344) | | | |
|---|---|---|---|---|---|---|---|---|
| | univariable | | multivariable | | univariable | | multivariable | |
| | OR (95%CI) | *p* | OR (95%CI) | *p* | OR (95%CI) | *p* | OR (95%CI) | *p* |
| Age (per year) | **1.18 (1.07, 1.29)** | **<.001**$^{***}$ | **1.20 (1.09, 1.32)** | **<.001**$^{***}$ | 0.94 (0.86, 1.03) | .173 | 0.95 (0.86, 1.04) | .269 |
| Sex (Female) | **0.71 (0.56, 0.90)** | **.005**$^{**}$ | **0.69 (0.54, 0.89)** | **.004**$^{**}$ | 0.86 (0.61, 1.20) | .366 | 0.81 (0.57, 1.16) | .253 |
| Gestational age (per week) | 1.03 (0.97, 1.08) | .354 | 0.93 (0.82, 1.06) | .271 | 1.00 (0.93, 1.08) | .919 | 0.98 (0.82, 1.18) | .842 |
| Birthweight z score (per SD) | 0.98 (0.89, 1.09) | .742 | 0.92 (0.76, 1.12) | .405 | 1.02 (0.88, 1.18) | .790 | 1.03 (0.78, 1.35) | .857 |
| Head circumference (per cm) | 1.03 (0.97, 1.10) | .268 | 1.04 (0.94, 1.15) | .460 | 1.01 (0.93, 1.10) | .735 | 0.99 (0.86, 1.15) | .936 |
| Multiple birth | 0.85 (0.65, 1.11) | .234 | 0.85 (0.64, 1.13) | .251 | 1.16 (0.77, 1.74) | .472 | 1.10 (0.73, 1.67) | .649 |
| IVH | 0.80 (0.61, 1.07) | .129 | 0.92 (0.67, 1.25) | .580 | 0.71 (0.48, 1.06) | .097 | 0.82 (0.54, 1.24) | .340 |
| BPD | **0.73 (0.55, 0.97)** | **.029**$^{*}$ | 0.79 (0.57, 1.08) | .144 | 0.81 (0.55, 1.20) | .303 | 0.87 (0.57, 1.33) | .513 |
| NSI | **0.39 (0.27, 0.54)** | **<.001**$^{***}$ | **0.37 (0.26, 0.53)** | **<.001**$^{***}$ | **0.43 (0.28, 0.65)** | **<.001**$^{***}$ | **0.44 (0.29, 0.69)** | **<.001**$^{***}$ |
| Higher maternal education | 0.89 (0.74, 1.06) | .196 | 0.84 (0.68, 1.04) | .117 | 0.91 (0.69, 1.20) | .514 | 0.88 (0.67, 1.16) | .371 |
| Maternal age at birth (per year) | 1.00 (0.98, 1.03) | .899 | 1.01 (0.99, 1.03) | .418 | 1.03 (1.00, 1.07) | .082 | 1.04 (1.00, 1.08) | .076 |
| Ethnicity (non-white) | **0.45 (0.29, 0.70)** | **<.001**$^{***}$ | **0.45 (0.29, 0.71)** | **.001**$^{**}$ | 0.58 (0.29, 1.18) | .131 | 0.57 (0.26, 1.26) | .175 |

| | **Work status (full-time vs. part-time)** (n=657) | | | | **Working hours per week** (n=656) | | | |
|---|---|---|---|---|---|---|---|---|
| | univariable | | multivariable | | univariable | | multivariable | |
| | OR (95%CI) | *p* | OR (95%CI) | *p* | B (95%CI) | *p* | B (95%CI) | *p* |
| Age (per year) | 1.07 (0.92, 1.25) | .386 | 1.06 (0.87, 1.29) | .571 | **0.96 (0.33, 1.60)** | **.008**$^{**}$ | **0.90 (0.28, 1.52)** | **.004** |
| Sex (Female) | **0.16 (0.10, 0.27)** | **<.001**$^{***}$ | **0.12 (0.07, 0.20)** | **<.001**$^{***}$ | **-5.73 (-7.09, -4.37)** | **<.001**$^{***}$ | **-6.15 (-7.55, -4.75)** | **<.001**$^{***}$ |
| Gestational age (per week) | 1.01 (0.93, 1.09) | .801 | 0.89 (0.73, 1.09) | .263 | 0.05 (-0.25, 0.36) | .728 | 0.08 (-0.63, 0.80) | .818 |
| Birthweight z score (per SD) | 0.94 (0.81, 1.10) | .450 | 0.78 (0.58, 1.04) | .086 | 0.06 (-0.50, 0.62) | .834 | 0.18 (-0.87, 1.23) | .742 |
| Head circumference (per cm) | 1.04 (0.95, 1.14) | .394 | 1.01 (0.87, 1.18) | .855 | 0.13 (-0.21, 0.47) | .457 | -0.26 (-0.82, 0.29) | .356 |
| Multiple birth | 1.02 (0.65, 1.59) | .944 | 1.09 (0.67, 1.78) | .737 | -1.16 (-2.82, 0.51) | .174 | -0.92 (-2.53, 0.68) | .261 |
| IVH | 0.73 (0.46, 1.17) | .191 | 0.86 (0.49, 1.53) | .613 | -1.36 (-3.19, 0.47) | .146 | -0.99 (-2.80, 0.82) | .285 |
| BPD | 0.70 (0.41, 1.20) | .199 | 0.60 (0.32, 1.15) | .124 | -1.70 (-3.64, 0.23) | .085 | -1.20 (-3.11, 0.71) | .219 |
| NSI | **0.40 (0.21, 0.74)** | **.004**$^{**}$ | **0.22 (0.11, 0.46)** | **<.001**$^{***}$ | **-3.72 (-6.22, -1.22)** | **.004**$^{**}$ | **-4.52 (-6.94, -2.1)** | **<.001**$^{***}$ |
| Higher maternal education | 0.96 (0.74, 1.25) | .771 | 0.95 (0.71, 1.28) | .734 | -0.11 (-1.11, 0.90) | .834 | 0.03 (-0.99, 1.05) | .954 |
| Maternal age at birth (per year) | 1.00 (0.96, 1.05) | .849 | 1.02 (0.97, 1.06) | .470 | -0.002 (-0.15, 0.15) | .975 | 0.03 (-0.11, 0.18) | .656 |
| Ethnicity (non-white) | 0.96 (0.45, 2.07) | .919 | 0.91 (0.39, 2.12) | .832 | -1.82 (-4.79, 1.16) | .232 | -1.25 (-4.67, 2.16) | .476 |

***Note.*** IVH = intraventricular haemorrhage, BPD = bronchopulmonary dysplasia, NSI = neurosensory impairments. $^{*}p < .05$, $^{**}p < .01$, $^{***}p < .001$.
